# Exploring the experiences, motivations, and skillsets of nurse volunteers during Hajj: implications for enhancing volunteer programs

**DOI:** 10.1186/s12912-024-01712-7

**Published:** 2024-01-16

**Authors:** Mashael Mohammed Alrashdi, Abdulellah Al Thobaity

**Affiliations:** 1 Ministry of Health, Riyadh First Health Complex Al Kharj Zone, 8295 Ibn Taqi, Al-Hamra district, Riyadh Region, Al-Kharj Governorate, Saudi Arabia; 2 Medical Surgical Nursing Department, College of Nursing, Taif University, 3966, 26516 Taif, Saudi Arabia

**Keywords:** Public health, Nurse volunteers, Hajj, Motivations, Experiences, Mass gathering

## Abstract

**Background:**

The annual Islamic pilgrimage of Hajj attracts millions of pilgrims from around the world, necessitating the involvement of healthcare professionals, including nurse volunteers, to ensure the safety and well-being of attendees. This study aims to explore the experiences, motivations, and skillsets of nurse volunteers during the Hajj pilgrimage, focusing on the personal, professional, and spiritual dimensions of volunteering, and providing insights to enhance volunteer programs.

**Methods:**

The demographic data shows that the majority of nurse volunteers during Hajj 2022 fell in the 30–39 age group and were predominantly women. The study provides insights into the interest, experience, and motives for volunteering, with spiritual motives cited as a primary driver. An overview of volunteer hours, perceived benefits, and pre-existing skills reveals diverse commitments and skillsets among the volunteers.

**Results:**

Nurse volunteers during Hajj 2022 show diverse age and gender composition, strong commitment, spiritual motives, varied engagement, and professional skills. Team dynamics, skills development, and positive experiences are crucial. A potential gap exists in preparatory education. Significant associations are found between age, volunteer hours, training, skill practice, and gender.

**Conclusion:**

This study highlights the multifaceted benefits of volunteering during Hajj and underscores the need for decision-makers to devise strategies that cater to diverse needs, foster collaboration, and enhance the preparedness of nurse volunteers. Further research is suggested to examine the long-term impact of volunteering during Hajj on nurses’ professional development and personal well-being and to evaluate the effectiveness of various recruitment strategies, training programs, and support initiatives.

## Introduction

The Hajj, one of the world’s largest mass gatherings, is an annual religious pilgrimage to the holy city of Makkah, Saudi Arabia, and represents one of the Five Pillars of Islam [1, 2]. With millions of Muslims participating from around the globe, the Hajj presents unique public health and safety challenges due to the sheer number of attendees and their close proximity during rituals [3]. Authorities in Saudi Arabia, including the Ministry of Health, invest substantial resources to manage the event, focusing on crowd control, infectious disease prevention, and medical service provision. As such, the Hajj highlights the significance of effective planning, coordination, and collaboration among various agencies and stakeholders to ensure the safety and well-being of all participants [4, 5].

During the Hajj, maintaining public health and safety is crucial given the convergence of millions of pilgrims [6]. To address these concerns, authorities implement various preventive and responsive measures aimed at minimizing health risks and ensuring attendees’ well-being. These measures include rigorous pre-arrival health screening, vaccination promotion, on-site robust medical facilities, and effective crowd management strategies [1, 7]. Health education and promotion campaigns also play a vital role in raising awareness among pilgrims about potential health hazards and preventive behaviors, such as maintaining personal hygiene and adhering to food safety guidelines [8]. By implementing comprehensive public health and safety measures, authorities strive to create a secure and healthy environment for all participants during the Hajj mass gathering.

Volunteering by healthcare providers, including nurses, is essential to ensuring the health and safety of the millions of pilgrims participating in the Hajj [7, 9]. Skilled healthcare volunteers address the increased demand for medical services and provide crucial support to existing healthcare infrastructure [3, 9]. Nurses and other healthcare professionals possess valuable expertise in areas such as triage, first aid, and patient care, enabling them to effectively manage health issues that may arise during the pilgrimage [10, 11]. Moreover, their background in health education and promotion equips them to inform and guide pilgrims on preventive measures and healthy practices, ultimately reducing disease transmission risks. Healthcare volunteers, including nurses, play a vital role in enhancing the overall quality of health services provided during the Hajj, safeguarding pilgrims’ well-being and promoting a safe and healthy pilgrimage experience for all [12].

The Saudi Ministry of Human Resources and Social Development reported that over 100,000 volunteers contributed to this year’s Hajj season, providing services in health, organization, and more. These efforts are part of the Kingdom’s Vision 2030 initiative. The volunteers’ contributions, recognized with a certificate, helped ensure a smooth and comfortable experience for the pilgrims [13]. The Health Volunteer Center at the Ministry of Health deployed 500 trained health volunteers to assist pilgrims, continuing their service from Ramadan, which benefited over 2 million people [14]. Challenges such as lack of skills and fear of cross-infections were identified, but despite these barriers, a significant majority of healthcare students expressed readiness to volunteer [15]. However, there is a lack of literature reporting on the experiences of volunteer nurses during Hajj, warranting further research in this area. In the context of nursing volunteers during Hajj, the study aims to explore the experiences of these volunteers, including aspects such as patient care, medical interventions, communication, cultural sensitivity, and working in a dynamic environment. The motivations being examined include religious duty, compassion for the pilgrims, personal growth, professional development, and community service. Examples of skillsets required for nursing volunteers during Hajj include emergency medical care, basic life support, managing health conditions and injuries, medication administration, effective communication, cultural competence, adaptability, and teamwork. By investigating these factors, the study seeks to gain insights into the role and contributions of nursing volunteers during Hajj. In the context of nursing volunteers during Hajj, the study aims to explore the experiences of these volunteers, including aspects such as patient care, medical interventions, communication, cultural sensitivity, and working in a dynamic environment. The motivations being examined include religious duty, compassion for the pilgrims, personal growth, professional development, and community service. Examples of skillsets required for nursing volunteers during Hajj include emergency medical care, basic life support, managing health conditions and injuries, medication administration, effective communication, cultural competence, adaptability, and teamwork. By investigating these factors, the study seeks to gain insights into the role and contributions of nursing volunteers during Hajj.

Investigating the experiences, motivations, and skillsets of nurse volunteers during the Hajj season in 2022 carries significant importance for several reasons. First, understanding their experiences and challenges can offer valuable insights into potential areas for improvement in healthcare service delivery during mass gatherings [16]. This knowledge can inform the development of targeted interventions and strategies to better support nurse volunteers in their roles. Second, examining their motivations can reveal the factors driving healthcare professionals to volunteer during the Hajj, which can be leveraged to encourage and sustain volunteerism in the future [17]. Finally, assessing nurse volunteers’ skillsets and identifying gaps in their knowledge and abilities can guide the design and implementation of tailored training programs, ultimately enhancing their competence and the quality of care provided to pilgrims. Conducting a cross-sectional analysis on these aspects offers an opportunity to gain a comprehensive understanding of the crucial role that nurse volunteers play during the Hajj season and inform evidence-based decisions for improving healthcare services in mass gatherings. Therefore, this study aimed to examine the Experiences, Motivations, and Skillsets of Nurse Volunteers.

## Methods

In this study, a cross-sectional descriptive design was employed to explore the experiences of nurse volunteers during the Hajj season in 2022. The researchers aimed to gain insights into the unique perspectives and challenges faced by Saudi nurses who served as volunteer health staff during the Hajj pilgrimage. To ensure the sample represented the target population accurately, purposive sampling was utilized. Purposive sampling ensured accurate representation of the target population by selecting participants based on specific criteria: prior experience as a Hajj volunteer nurse, professional qualifications, and availability during the research period. This strategy ensured that the sample closely mirrored the characteristics and experiences of the broader population of Hajj nurse volunteers. The study was conducted within the setting of the Hajj pilgrimage in Saudi Arabia, where millions of pilgrims gather each year. This context provided a unique environment for nurse volunteers, characterized by a large influx of individuals requiring healthcare services and the need for effective coordination and provision of care. Ethical approval for the study was obtained from the Institutional Review Board (IRB) at KACST, KSA, with the IRB registration number HAP-02-T-067, approval number 721, and approval date of 15/08/2022.

Data collection for the study occurred after the Hajj season of 2022. The study specifically focuses on nurses who volunteered during the Hajj season of 2020 as the criteria for participation. From August 20, 2022, to September 20, 2022, the researchers distributed a self-administered questionnaire to a selected sample of Saudi nurses who participated as volunteer health staff. Participants were approached and invited to participate by compiling a list of nurses who volunteered during the Hajj season of 2020, followed by contacting them through phone calls or emails to explain the study’s purpose, benefits, and expected commitment. Participants had the opportunity to ask questions and were provided with informed consent forms upon expressing consent. The recruitment process ensured transparency and ethical practices for willing participation. Approximately 300 volunteer nurses were invited to participate in the study. Validity and reliability were key measures in this study, with face validity and content validity assessed by three experts in the field for clarity and relevance. These experts were highly qualified, each holding a PhD in Nursing. Their professional experience and academic expertise were further underscored by their positions as associate professors and assistant professors, respectively. This ensured a comprehensive and rigorous evaluation of the study’s validity. Reliability was measured using Cronbach’s alpha which was 0.70 indicating a satisfactory level of reliability for the scales used in the study. An explanation statement was obtained from all participants prior to administering the questionnaire in the study. This statement served as a written document that clearly explained the research project’s purpose, procedures, and expectations to the participants. It included information about the study’s objectives, data collection process, potential risks and benefits, confidentiality measures, and other relevant details. By ensuring that participants had access to this comprehensive information, they were able to make informed decisions about voluntarily participating in the research. Obtaining an explanation statement was a standard ethical practice that promoted transparency and protected the participants’ rights and well-being.

For data analysis, descriptive statistics were used to understand the distribution of participants’ demographic and background information, such as age, gender, interest, experience, information sources, motives, volunteer hours, perceived benefits, pre-existing skills, team dynamics, and participation in pre-volunteering training courses and practical skills workshops. Frequencies and percentages were calculated to summarize the categorical data, which helped identify the most common characteristics and patterns among the participants. Additionally, to measure the relationship between various categorical variables related to volunteering, the analysis methods employed include the Chi-Square Test and Cramer’s V. The Chi-Square Test is a statistical test used to determine the association between categorical variables, while Cramer’s V quantifies the strength of the relationship between those variables. The variables analyzed encompass age distribution, gender distribution, volunteering interest, volunteering experience, average volunteer hours, training courses and skill practice perception, motives for volunteering, and returns received.

## Results

Table 1 in the study of nurse volunteers during Hajj 2022 provides an overview of the participants’ age and gender distribution. A significant portion (65.7%) belonged to the 30–39 age group, indicating that the majority of volunteers were in their early to mid-career stage. The representation of younger professionals in the 20–29 age group (17.1%) and the smaller proportion of volunteers in the 40–49 age group (12.6%) and those over 50 years old (4.0%) suggest a diverse age composition among participants. In terms of gender distribution, a substantial majority of volunteers were female (72.0%), while 27.4% were male, reflecting the broader trend of a predominantly female workforce in the nursing profession. Overall, the descriptive statistics in Table 1 offer valuable insights into the demographics of nurse volunteers during Hajj 2022, which can inform future recruitment and training strategies in similar contexts.

**Table 1:** Age and gender distribution of nurse volunteers during Hajj 2022

Categories		Frequency	Percent
Age	20–29	30	17.24
	30–39	115	66.09
	40–49	22	12.65
	More than 50	7	4.02
	Total	174	100
sex	Male	48	27.59
	Female	126	72.41
	Total	174	100

Table 2 in the study of nurse volunteers during Hajj 2022 reveals insights into their interest, experience, information sources, and motives for volunteering. A majority (65.1%) consistently expressed interest, indicating a strong commitment to volunteering, while 68.0% had prior experience, suggesting familiarity with the responsibilities involved. The table also highlights the significance of diverse communication channels, with 45.1% learning about opportunities through social media, 32.6% from their workplace, and 22.3% from health volunteer platforms. These findings underscore the importance of effective dissemination of information about volunteering opportunities. Furthermore, spiritual motives were cited as the primary driver for volunteering by 50.3% of participants, reflecting a strong spiritual connection and desire to serve their community during this significant religious event. Cognitive motives (28.6%) and material motives (21.1%) also played a role, demonstrating that a combination of factors influenced the decision to volunteer during Hajj 2022.

**Table 2:** Interest, experience, information sources, and motives of nurse volunteers during Hajj 2022

Categories		Frequency	Percent
Are you interested in volunteering?	Yes	114	65.42
	Sometimes	53	30.56
	No	7	4.02
	Total	174	100
Have you ever volunteered before?	Yes	119	68.0
	No	54	30.9
	Total	173	98.9
How did you get news and information about volunteer opportunities for Hajj 2022?	From workplace	57	32.6
	from social media	79	45.1
	Health volunteer platforms	39	22.3
	Total	175	100.0
What were the motives that made you go to volunteer in Hajj 2022?	Cognitive motives	50	28.6
	Material motives	37	21.1
	spiritual motives	88	50.3
	Total	175	100.0

Table 3 in the study of nurse volunteers during Hajj 2022 highlights the diversity in time commitment, perceived benefits, and pre-existing administrative and clinical skills. A majority (60.6%) volunteered for 10–20 h, indicating varied levels of engagement among participants. The primary perceived returns included developing acquired skills (62.3%), building social relationships (32.0%), and spending spare time productively (5.7%), emphasizing the multifaceted benefits of volunteering. Furthermore, the participants reported a wide range of administrative and clinical skills prior to their involvement in Hajj 2022. Nearly half (46.9%) possessed a combination of leadership, management, coordination, and rapid response skills in emergency situations, while 49.1% had expertise in needle technique, intravenous solutions, cardiopulmonary resuscitation, and wound dressing. These diverse skillsets reflect the professional background and capabilities of the nurse volunteers, which can contribute to the overall success and effectiveness of the volunteer program during Hajj 2022.

**Table 3:** Volunteer hours, returns, and pre-existing skills of nurse volunteers during Hajj 2022”

Categories		Frequency	Percent
What was the average number of hours you dedicated to volunteering during Hajj 2022?	10–20 h	106	60.6
	21–30 h	26	14.9
	31–40 h	23	13.1
	More than 40 h	20	11.4
	Total	175	100.0
What benefits did you receive from your participation in Hajj 2022?	Develop acquired skills	109	62.3
	Building social relationships	56	32.0
	spending spare time	10	5.7
	Total	175	100.0
Which administrative skills did you have prior to your involvement in Hajj 2022?	leadership and management	24	13.7
	Coordination	42	24.0
	rapid response in emergency situations	27	15.4
	All of the above	82	46.9
	Total	175	100.0
What clinical expertise did you possess before participating in Hajj 2022?	Needle technician and intravenous solutions	29	16.6
	Cardiopulmonary resuscitation technician	19	10.9
	Wound dressing technician	41	23.4
	All the above	86	49.1
	Total	175	100.0

Table 4 in the study of nurse volunteers during Hajj 2022 explores the role of social support, team dynamics, skills development, and overall volunteer experience. A majority of participants (63.4%) found it helpful to have people they knew participating with them in Hajj 2022, highlighting the importance of familiar connections during the event. In terms of the most crucial aspect for a volunteer, 44.6% considered the team as the most important, followed by individual roles and tasks (29.7%) and the leader (25.7%), emphasizing the role of teamwork and personal responsibilities.

**Table 4:** Social support, team dynamics, skills development, and volunteer experience during Hajj 2022”

Categories		Frequency	Percent
Did having acquaintances participate alongside you in Hajj 2022 prove beneficial?	Yes	111	63.4
	No	35	20.0
	May be	29	16.6
	Total	175	100.0
In your view, which aspect was most crucial for volunteers during your involvement in Hajj 2022?	The leader	45	25.7
	Team	78	44.6
	your roles and tasks in the team	52	29.7
	Total	175	100.0
Did applying your skills make your experience in Hajj 2022 distinct?	Yes	137	78.3
	No	14	8.0
	May be	24	13.7
	Total	175	100.0
Based on your volunteer experience during Hajj 2022, are you encouraged to participate again?	Yes	138	78.9
	No	9	5.1
	May be	28	16.0
	Total	175	100.0
Did your supervisor interact with you in a flexible and comfortable manner during Hajj 2022?	Yes	125	71.4
	No	13	7.4
	May be	37	21.1
	Total	175	100.0
When you came across a new skill while volunteering in Hajj 2022, did you inquire about it?	Yes	132	75.4
	No	19	10.9
	May be	24	13.7
	Total	175	100.0

A significant proportion of participants (78.3%) reported that practicing their skills made a difference in their experience during Hajj 2022, demonstrating the value of applying professional expertise in a volunteer setting. A majority (78.9%) found their experience in Hajj 2022 encouraging enough to consider repeating it, indicating a positive overall experience. Most participants (71.4%) experienced flexible and comfortable interactions with their supervisors, contributing to a supportive work environment. Lastly, when encountering a new skill during their volunteering, 75.4% of participants sought information about it, reflecting an eagerness to learn and develop professionally during Hajj 2022. This table underscores the significance of social support, teamwork, skills development, and positive experiences in fostering a successful volunteer program.

Table 5 in the study of nurse volunteers during Hajj 2022 examines the provision of training courses and practical skills workshops before participating in the event. Less than half of the participants (44.6%) reported receiving training courses prior to volunteering for Hajj 2022, indicating a potential gap in preparatory education. Regarding the practical skills workshops, 41.7% of the nurse volunteers participated in such workshops before their involvement in Hajj 2022, while 42.3% did not, and 16.0% were uncertain. The data highlights the varying levels of preparatory training and workshops provided to the volunteers, suggesting a need for more consistent and comprehensive training opportunities to ensure their readiness and effectiveness during events like Hajj 2022.

**Table 5:** Pre-volunteering training courses and practical skills workshops for nurse volunteers in Hajj 2022

Categories		Frequency	Percent
Were you provided with training courses prior to volunteering for Hajj 2022?	Yes	78	44.6
	No	97	55.4
	Total	175	100.0
Did you participate in a practical skills workshop before your involvement in Hajj 2022?	Yes	73	41.7
	No	74	42.3
	May be	28	16.0
	Total	175	100.0

Table 6 summarizes the associations between demographic factors, volunteering interest, experience, and motives among nurse volunteers in Hajj 2022. The findings show no significant associations between age, gender, and volunteering interest and experience. However, there are significant associations between age group and average volunteer hours for Hajj 2022, and between training courses and the perception of skill practice. The motives for volunteering are significantly associated with gender but not with other factors analyzed.

**Table 6:** Associations between Demographic Factors, Volunteering Interest, Experience, and Motives in Hajj 2022

Factors	Association	Chi-Square Test *p*-value	Cramer’s V
Age distribution and volunteering interest	No significant association	0.825	0.091
Gender distribution and volunteering interest and experience	No significant association	0.310	0.116
Age and volunteering experience	No significant association	0.791	0.077
Gender and volunteering experience	No significant association	0.113	0.120
Gender and average volunteer hours for Hajj 2022	No significant association	0.062	0.205
Age group and average volunteer hours for Hajj 2022	Significant association	0.006	0.210
Training courses and skill practice perception	Significant association	0.009	0.231
Motives for volunteering and age group	No significant association	0.210	0.155
Motives for volunteering and interest in volunteering	No significant association	0.162	0.137
Motives for volunteering and previous volunteering experience	No significant association	0.645	0.071
Motives for volunteering and average volunteer hours for Hajj 2022	No significant association	0.515	0.122
Motives for volunteering and returns received in Hajj 2022	No significant association	0.471	0.101
Motives for volunteering and gender	Significant association	0.004	0.250

## Discussion

Volunteering during Hajj is of paramount importance for ensuring the smooth functioning of the annual Islamic pilgrimage, which attracts millions of pilgrims from around the world. Volunteers contribute in various ways, including providing healthcare services, managing crowds, ensuring sanitation, facilitating transportation, and addressing the diverse needs of the pilgrims [18, 19]. For nurses, volunteering during Hajj offers a unique opportunity to serve others, strengthen their faith, and gain invaluable professional experience in a challenging environment. This study highlights the multifaceted motivations of nurses to volunteer during Hajj, encompassing personal, professional, and spiritual dimensions. Volunteering helps deepen their spiritual connection, fosters a sense of community, and provides opportunities for networking and skill development [20]. Interacting with healthcare professionals from diverse backgrounds promotes the exchange of knowledge and best practices, facilitating professional growth and equipping nurses with new skills and insights into global healthcare challenges. The demographic analysis of nurse volunteers during Hajj 2022 reveals a substantial participation from professionals in their early to mid-careers, primarily aged between 30 and 39, possibly due to the physically demanding nature of the event, career advancement opportunities, or religious and philanthropic inclinations. A significantly lower representation from the younger (20–29) and older age groups (40 and above) necessitates further investigation into whether younger nurses are more inclined to volunteer or if they have more opportunities. In terms of gender, there’s a predominance of female volunteers, reflecting the broader trend in the nursing profession. Thus, strategies to enhance volunteers’ recruitment and training should consider these demographics, emphasizing skill development and career progression, fostering inclusivity, and catering to diverse needs and motivations [21–23]. Regular evaluations and feedback collection are also crucial to refine these strategies and improve support mechanisms [21–23]. To improve recruitment strategies, decision-makers should focus on underrepresented age groups or gender demographics, aiming for a diverse and balanced volunteer representation. This may involve initiating outreach initiatives or forming partnerships with organizations catering to specific age groups or genders. To further increase the reach of recruitment efforts, employ various communication channels, including social media, workplace announcements, and health volunteer platforms, to efficiently disseminate information about volunteer opportunities and engage a wider audience [23–25].

A significant majority of the volunteers, reported at 65.1%, consistently expressed an interest in volunteering. This strong commitment to volunteering indicates a deep-seated desire to serve and contribute, potentially driven by personal satisfaction, professional development, or humanitarian concerns. Another noteworthy finding is that 68.0% of the nurse volunteers had previous volunteering experience. This suggests that these volunteers are well-acquainted with the responsibilities and challenges that come with volunteering. It could also indicate a sense of fulfillment or positive experiences in their past volunteering roles, leading them to volunteer again. The study also highlights the vital role of various communication channels in providing information about volunteering opportunities. The most effective among these was social media, with 45.1% of the nurses learning about the opportunities through this medium Periodically updating these channels with pertinent information can sustain interest and promote participation [25]. This underlines the power and reach of digital platforms in today’s connected world. Workplaces also served as significant sources of information, accounting for 32.6% of the volunteers, while health volunteer platforms attracted 22.3% of volunteers. These data points stress the importance of leveraging multiple platforms to effectively disseminate information and attract potential volunteers. In terms of motivation, spiritual reasons were cited as the primary driver for volunteering by over half of the participants (50.3%). This reflects the deep spiritual ties of the event and the desire of the volunteers to serve their community during such an important religious occasion. Cognitive (28.6%) and material (21.1%) motivations also played significant roles, showing that the decision to volunteer is often influenced by a combination of factors. These might include the desire to learn and develop new skills, the opportunity to gain professional experience, or the availability of certain incentives or rewards. Address various volunteering motives, such as community, cognitive, and material motives, by highlighting the multifaceted benefits of volunteering and offering incentives or recognition for volunteers’ efforts [26, 27]. This approach can help sustain engagement and commitment among volunteers with diverse motivations. Offer flexible scheduling and accommodations that cater to the diverse needs of volunteers across age groups and genders [22, 28]. This can encompass part-time or full-time opportunities, age-sensitive accommodations, and gender-specific facilities and resources. Conducting comparative studies to explore the experiences, motivations, and skillsets of nurse volunteers in other large-scale events or disaster settings can provide valuable insights into the diverse contexts in which nurses volunteer.

Volunteering plays a crucial role in our society, offering a range of benefits to those who participate [15, 27]. A recent survey revealed that most volunteers (60.6%) commit between 10 and 20 h, indicating a moderate level of engagement. The primary benefits of volunteering, as perceived by the participants, included skill development (62.3%), fostering social relationships (32.0%), and productive use of spare time (5.7%). These findings underscore the multifaceted advantages of volunteering, offering insights into how it allows individuals to grow personally and professionally, build social connections, and utilize their time in a productive manner. That to say, volunteering serves as a crucial catalyst for personal growth, societal development, and social connectivity [3, 29]. By fostering skill development, enhancing social relationships, and providing a productive avenue for spare time, it presents a unique platform for individuals to grow and contribute meaningfully to their communities [23, 30]. Encouraging volunteering thus promises a more engaged, skillful, and interconnected society, enriching individuals’ lives and the wider community in manifold ways [29, 31]. The recent survey indicates a diverse range of pre-existing skills among volunteers in the Hajj 2022 program. With 46.9% possessing leadership, management, coordination, and emergency response skills, and 49.1% demonstrating proficiency in technical medical skills like needle technique, intravenous solutions, CPR, and wound dressing, the volunteers contribute significantly to the operational efficiency and emergency preparedness of the event. This diversity in skill sets, from administrative to clinical, not only showcases the high professional capabilities of the volunteers but also underscores their potential to enhance the success of the volunteer program during large-scale events like Hajj [32–34].

Less than half of the participants (44.6%) reported receiving training courses prior to their involvement in Hajj 2022. This suggests that there may be a significant gap in the provision of preparatory education for nurse volunteers. The reasons for this gap and the potential implications on the volunteers’ effectiveness during Hajj could be explored. comprehensive training courses and practical skill workshops should be provided to all volunteers before participating in events like Hajj. This study reveals that only 41.7% of the nurse volunteers participated in practical skills workshops before their involvement in Hajj 2022, while 42.3% did not, and 16.0% were uncertain. This suggests a lack of consistency in the provision of such workshops. The reasons for this inconsistency and its potential impact on the quality of service provided by the volunteers could be examined. These sessions must be accessible, relevant, and tailored to the unique needs and backgrounds of the volunteers, thereby enhancing their preparedness and effectiveness during the event. It is noteworthy that 16.0% of participants were uncertain about whether they participated in practical skills workshops. This indicates a potential issue with communication or record-keeping, which could be another point of discussion. Therefore, establishing mentorship and support networks for inexperienced volunteers or those seeking to develop new skills during their volunteering experience is crucial [25]. Implementing a mentorship program or buddy system that pairs seasoned volunteers with novices can facilitate knowledge sharing and support. Cultivating a robust sense of teamwork and collaboration among volunteers is essential. This can be achieved by emphasizing the significance of team dynamics and personal responsibilities in training and during the event [27, 35]. Promote team-building activities and knowledge sharing to create a supportive work environment where volunteers feel connected and engaged [27, 35].

Emphasizing the importance of team dynamics and personal responsibilities during training sessions is also key to cultivating a strong sense of teamwork and collaboration among volunteers. As for further research, investigating the long-term impact of volunteering during Hajj on nurses’ professional development, career trajectory, and personal well-being can help better understand the benefits and challenges associated with such experiences [36, 37]. Evaluating the effectiveness of various recruitment strategies, training programs, and support initiatives in enhancing the preparedness, performance, and satisfaction of nurse volunteers during events like Hajj can help identify best practices and inform future strategies to optimize the volunteer experience.

To create a more inclusive and diverse volunteer force, it is important to not only focus on recruitment strategies but also on retention efforts. This can be achieved by recognizing the contributions of volunteers, celebrating their achievements, and providing opportunities for personal and professional growth [38, 39]. Encourage feedback and open communication between volunteers and organizers to ensure a positive and fulfilling experience for all involved [40]. In the context of Hajj, understanding the cultural and religious aspects of volunteering is essential. This insight can help tailor training programs and support initiatives to meet the specific needs of Muslim nurse volunteers, thereby increasing their effectiveness and satisfaction during the event [27, 41]. Similarly, incorporating cultural and religious considerations into recruitment strategies can help attract a diverse pool of volunteers who are motivated and committed to serving their community during this significant religious event.

Moreover, collaboration between various stakeholders, including government agencies, non-governmental organizations, and health institutions, is crucial for the successful implementation of volunteer programs during events like Hajj [42, 43]. By working together, these entities can pool resources, share expertise, and streamline processes to ensure the well-being of both volunteers and pilgrims [44]. Establishing a centralized system for coordinating volunteer efforts can also help facilitate communication, training, and resource allocation among different organizations and sectors involved in Hajj [44]. Finally, it is important to recognize that the challenges and opportunities faced by nurse volunteers during Hajj are not limited to this specific event. Large-scale religious gatherings, mass events, and disaster relief efforts all require the involvement of dedicated and skilled nurse volunteers [39, 43, 45]. By learning from the experiences of Hajj volunteers, researchers and practitioners can develop strategies to improve volunteer programs in various contexts, ultimately benefiting the broader healthcare community and the individuals they serve.

## Conclusion

In conclusion, this study underlines the significant role of healthcare volunteers, especially nurses, in ensuring the smooth execution of large events like the Hajj. The findings highlight that personal satisfaction, professional development, and spiritual fulfillment are key motivators for volunteer engagement. However, gaps in training and preparation, as well as inconsistencies in the provision of practical skill workshops, suggest areas for improvement. To optimize volunteer readiness and performance, comprehensive training and practical skill workshops tailored to the volunteers’ needs should be provided. Future research should delve into the long-term effects of volunteering on professional development and personal well-being, and the effectiveness of recruitment and training programs. Understanding the cultural and religious aspects of volunteering during Hajj can also help to tailor these initiatives. The insights gained from this study can inform volunteer programs in various contexts, ultimately benefiting the broader healthcare community.

### Limitation

Our study has several limitations. The cross-sectional design provides a snapshot and not a longitudinal view. The self-reported data might suffer from response and recall bias. Cultural factors specific to Hajj pilgrimage might limit the applicability of results to other settings. The sample comprised only Saudi nurses, possibly not reflecting experiences of other health professionals.

## Data Availability

The datasets generated and/or analyzed during the current study are available from the corresponding author upon reasonable request.
